# Hierarchical Compositional Alignment for Zero-Shot Part-Level Segmentation

**DOI:** 10.3390/s26072130

**Published:** 2026-03-30

**Authors:** Shan Yang, Shujie Ji, Zhendong Xiao, Xiongding Liu, Wu Wei

**Affiliations:** 1 School of Automation Science and Engineering, South China University of Technology, Guangzhou 510640, China; 2 School of Automation, Hangzhou Dianzi University, Hangzhou 310018, China

**Keywords:** visual language models, part segmentation, multi-hierarchy feature, multimodal alignment

## Abstract

In robotic fine-grained tasks (e.g., grasping and assembly), precise interaction requires a detailed understanding of object components. While Visual Language Models (VLMs) excel at object-level recognition, they struggle with part-level segmentation (e.g., knife handles), limiting performance in complex scenarios. VLMs face three key challenges: (1) Visual granularity mismatch—object-level features lack part-level details; (2) Semantic hierarchy gaps—parts and objects differ significantly in semantics; (3) Cross-modal bias—CLIP’s text–image alignment favors global over local features. To address these, we propose a one-stage VLM-based part segmentation method. First, the Hierarchy-Aware Feature Selection mechanism analyzes Transformer features in different hierarchies to enhance spatial and semantic precision for part segmentation. Second, the Multi-Hierarchy Feature Adapter bridges object-to-part feature granularity via the hierarchical adaptation. Finally, the Hierarchical Multimodal Alignment Module harmonizes classification accuracy and mask integrity via hierarchical alignment of vision–language, mitigating the bias of CLIP’s object-level priori knowledge. Experiments show the proposed method improves part segmentation performance for Zero-Shot, achieving 25.86% on Pascal-Part and 13.09% on ADE20K-Part (gains of +0.81% hIoU and +2.96% hIoU over baseline). This work advances robotic visual perception, with applications in intelligent manufacturing and intelligent service.

## 1. Introduction

An embodied intelligent agent is an interactive system that communicates with humans through its perceptual capabilities, interacts with the environment, makes decisions, and takes actions using human-like working and reasoning methods [1]. Object-level visual tasks currently limit an agent’s environmental perception, consequently impairing its decision-making and actions. In such tasks, agents can only recognize whole objects rather than parts. Part-aware perception becomes critical in operational scenarios—for instance, when preparing food, we rely on specific parts of utensils (e.g., a knife’s handle is used for the robot to cut vegetables, or a spatula’s handle is convenient for the robot to stir). Thus, the ability to recognize and reason about object parts is essential for an agent’s perceptual system, holding significant application value in industrial domains, particularly robotics [2,3,4,5].

However, part-level semantic segmentation requires large-scale datasets with detailed part annotations. While existing public datasets like ADE20K-Part [6], Pascal-Part [7] provide such annotations, they suffer from two critical limitations: (1) the covered object categories remain significantly limited compared to real-world diversity, and (2) the part taxonomy becomes inherently more complex, as each object typically comprises multiple constituent parts. Therefore, when applying models trained on public datasets for testing, they inevitably encounter unseen part categories. To address this challenge, researchers have proposed a new paradigm called Zero-Shot Semantic Segmentation (ZSS) [8,9,10,11]. This paradigm trains models on seen classes, enabling them to generalize to unseen classes effectively. The rapid development of Zero-Shot semantic segmentation has benefited from advances in Open Vocabulary Semantic Segmentation (OVSS) [12,13,14,15,16], particularly through visual language models (VLMs) like CLIP [17].

Despite OVSS excelling at object-level tasks, it faces additional challenges at the part level [3]. Parts exhibit more complex structures than objects, typically featuring intricate boundaries and greater appearance variations. Moreover, compared to objects, annotated training data for part segmentation remains significantly scarce [6,7]. The open-vocabulary setting introduces further challenges that need to be addressed. While objects are typically well-defined entities with clear boundaries, parts exhibit flexible granularity, introducing open-granularity challenges rarely seen in object-level OVSS [12]. Furthermore, prevailing large-scale VLMs are pre-trained on natural image–text pairs emphasizing object-level concepts, inherently limiting their part recognition capability [18].

Considering the aforementioned challenges, we address the OVSS problem for complex part datasets by leveraging the shared semantic features of common object parts to classify parts of unseen objects. Across different objects, parts such as the legs, torso, and head of quadrupedal animals (e.g., cats, dogs, and elephants) exhibit similar semantic characteristics. Within the same object category, taking quadrupedal animals as an example, their forelegs, hind legs, left legs, and right legs also demonstrate comparable semantic features. Therefore, we leverage this part-level semantic feature similarity to enhance data efficiency in part-based OVSS. Our findings demonstrate that the semantic features extracted by models significantly influence their part-level OVSS capability. To address the lack of part-aware feature extraction in pre-trained models, we propose the Multi-Hierarchy Feature Adapter (MHFA) that shifts the model’s attention from object-level to part-level representations. Within our one-stage segmentation framework, we investigate the Hierarchical Multimodal Alignment Module (HMAM) to enable textual features to guide part segmentation. Furthermore, we evaluate the Zero-Shot part segmentation performance on both Pascal-Part and ADE20K-Part datasets.

This work proposes a one-stage VLM-based segmentation approach that systematically addresses three core challenges in part segmentation. First, the Hierarchy-Aware Feature Selection (HAFS) mechanism effectively bridges the visual granularity gap between object-level and part-level representations. Second, the Multi-Hierarchy Feature Adapter (MHFA) enables progressive cross-granularity feature transformation through an adapter mechanism. Finally, the proposed Hierarchical Multimodal Alignment Module (HMAM) rectifies CLIP’s global alignment bias. This approach enhances fine-grained segmentation performance while preserving VLM’s Zero-Shot capabilities, offering a novel technical solution for applications requiring part-level understanding, such as robotic precision manipulation.

## 2. Related Works

### 2.1. Multi-Hierarchy Feature Fusion

Multi-scale features have been widely demonstrated to significantly enhance performance in object-level segmentation and detection tasks [19,20]. The commonly used multi-hierarchy feature fusion methods include Unet [21,22,23], ADD [24], FPN [25,26], and Attention [18,21,27], among others. VLPart [18] employs FPN (Feature Pyramid Network) to fuse features from the third, sixth, and ninth layers of ResNet. CLIPSeg [28] adopts a direct concatenation approach to integrate features from the third, sixth, and seventh layers of its Transformer architecture [29]. Atlas [30] utilizes cross-attention mechanisms to enable effective information exchange across multiple windows and hierarchy. SegLD [31] implements a diffusion model as its image encoder, where the noise-adding and denoising processes inherently fuse multi-hierarchy features through the U-Net structure to generate comprehensive image representations. This study investigates the impact of multi-scale feature fusion on part segmentation performance.

### 2.2. Multi-Modal Feature Alignment

In vision–language model research, multimodal alignment has proven to be critically important, as it enhances model performance on various cross-modal downstream tasks, including semantic segmentation, object detection, and classification. For classification tasks, both CLIP and ZegFormer employ contrastive learning to align text and image embeddings, though they derive image embeddings from different sources. CLIP [17] is a dual-encoder model with a Vision Transformer image encoder and a Transformer text encoder, pre-trained contrastively on 400M image–text pairs to align images and texts in a shared embedding space. In semantic segmentation, CLIPSeg [28] introduces the Feature-level Image–text Mutual Integration (FIMI) module to fuse multimodal features through multiplicative and additive operations between the two modalities.

Hierarchical Open-vocabulary Universal Image Segmentation [32] utilizes Bi-cross-Attention to effectively fuse text and image features through bidirectional cross-modal interaction. Meanwhile, both ZegCLIP [33] and ZegOT [34] generate Relationship Descriptors by performing element-wise multiplication between global visual embeddings and text embeddings, followed by concatenation with the original text embeddings, which are then integrated with image features via cross-attention mechanisms. In contrast, Fusioner [35] adopts a distinct approach where image features and text embeddings are first concatenated before undergoing self-attention computation through Transformer layers. Building upon these diverse methodologies, this work systematically investigates various text–image alignment strategies to identify the most effective approach for multi-scale feature–text fusion in part segmentation tasks, with particular emphasis on optimizing the alignment between hierarchical visual features and linguistic representations.

### 2.3. Semantic Segmentation of Vision–Language Models

In VLMs based semantic segmentation, existing approaches can be broadly categorized into one-stage and two-stage paradigms. Two-stage models typically consist of: (1) a proposal generation stage for extracting region candidates, followed by (2) a classification prediction stage for category assignment and segmentation refinement [13,18,36]. In two-stage models, the proposal generation stage predominantly incorporates MaskFormer-based architectures, as exemplified by ZSeg, VLPart, and PartSeg. Furthermore, certain two-stage variants [37] build upon the Segment Anything Model (SAM); while SAM inherently generates category-agnostic masks, these approaches append a subsequent category prediction stage to assign class labels to the produced masks. The category prediction stage performs similarity computation between the image features extracted during the proposal generation stage and the text features to determine the class labels for corresponding regions. Alternatively, some two-stage models [15] employ diffusion models to simultaneously generate both segmentation masks and their corresponding mask embeddings, which are subsequently fed into a text classifier for category prediction. Notably, SAM-CLIP [38] adopts a cascaded approach where CLIP first produces coarse masks serving as prompts for SAM, which then refines these masks through its sophisticated decoding architecture.

One-stage models are end-to-end architectures that directly generate category-aware masks [28,33]. In CLIPSeg [28], the output masks inherently contain class information, typically represented through different channels in the final output layer, where each channel corresponds to a specific category, and pixels belonging to the same class are grouped within the same channel. Unlike two-stage approaches that perform regional similarity computation, one-stage models calculate feature similarity between the entire image’s visual features and text embeddings globally. ZegCLIP [33] enhances CLIP’s architecture through Deep Prompt Tuning, which strategically optimizes the image encoder while preserving CLIP’s generalized representation capabilities. This adaptation produces image features better suited for semantic segmentation tasks. The model then generates class-specific masks by performing Multi-Head Attention via alignment information between class token embedding of the image encoder, text embedding as the Query, and the image features after Deep Prompt Tuning as the Key and Value.

The field of vision–language segmentation has evolved beyond traditional one-stage and two-stage models to include hybrid architectures. OVSeg [39] employs a three-stage framework. The process first utilizes MaskFormer to generate class-agnostic mask proposals and their corresponding embeddings, then it crops the original image based on these proposals to obtain region-specific sub-images. The final stage computes dual similarity measures between text embeddings and both the cropped sub-images and mask proposals, combining these through a learned weighting mechanism to produce the final classification results.

The two-stage model’s reliance on class-agnostic proposal networks presents fundamental limitations for open-granularity scenarios, as these proposal networks are typically trained on predefined part categories. Furthermore, mask proposal models demonstrate a strong tendency to overfit to their training data, significantly compromising their Zero-Shot generalization capability. These critical shortcomings motivate our adoption of a one-stage architecture for Zero-Shot part segmentation tasks.

## 3. Methods

**Figure 1:**
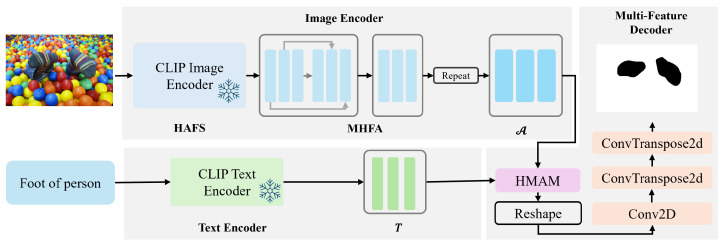
The framework of Hierarchy-Aware Vision–Language Alignment for Zero-Shot Part Segmentation. The process is decomposed into three steps: (1) the image encoder extracts multi-scale image features; (2) the text encoder extracts text features; and (3) the part segmentation generation module fuses image–text features and generates part masks.

Figure 1 illustrates the framework of Hierarchy-Aware Vision–Language Alignment for Zero-Shot Part Segmentation, which mainly comprises three core components: Hierarchy-Aware Feature Selection (HAFS), Multi-Hierarchy Feature Adapter (MHFA) and Hierarchical Multimodal Alignment Module (HMAM). HAFS utilizes attention scores to analyze different hierarchical features from the Clip Image Encoder, exploring where multi-grained features pay greater attention to details. MHFA employs an adapter to fine-tune the parameters of the Clip Encoder, extracting suitable features for part-level segmentation. HMAM aligns image features at different hierarchical levels with text embeddings, respectively, and fuses these multi-level features to ensure both classification accuracy and shape integrity of the masks.

### 3.1. Hierarchy-Aware Feature Selection

Inspired by CLIPSeg [28] and OVseg [12], we posit that different hierarchical feature layers in a Transformer encoder capture distinct levels of visual detail. The image encoder adopts a ViT architecture comprising 12 blocks, consistent with CLIPSeg. While object-level tasks typically utilize features from the final (12th) block, we find this approach insufficient for part-level segmentation, leading to significantly degraded performance. To address this, we propose a Hierarchy-Aware Feature Selection (HAFS) module that identifies optimal feature representations by analyzing attention scores across different layers. Specifically, HAFS analyzes the self-attention maps (Equation (1)) generated across transformer blocks. These maps, derived from the SoftMax-normalized dot products of queries and keys (Equation (2)), reveal how the model allocates its focus across spatial dimensions. Higher scores within an attention map indicate the model’s stronger focus on the corresponding spatial features. Our method leverages this by extracting a set of positionally aware, multi-level features from these attention-rich regions, which are critically important for segmenting fine-grained parts.

(1) $$\mathrm{Attention}\left(Q,K,V\right)=\mathrm{softmax}\left(\frac{QK^{T}}{\sqrt{d_{k}}}\right)V$$

(2) $$AttentionScore=softmax(\frac{QK^{T}}{\sqrt{d_{k}}})$$

where *Q* (Query), *K* (Key), and *V* (Value) are the input matrices of the self-attention mechanism, $K^{T}$ denotes the transpose of *K*, and $d_{k}$ is the dimensionality of the key vectors.

**Figure 2:**
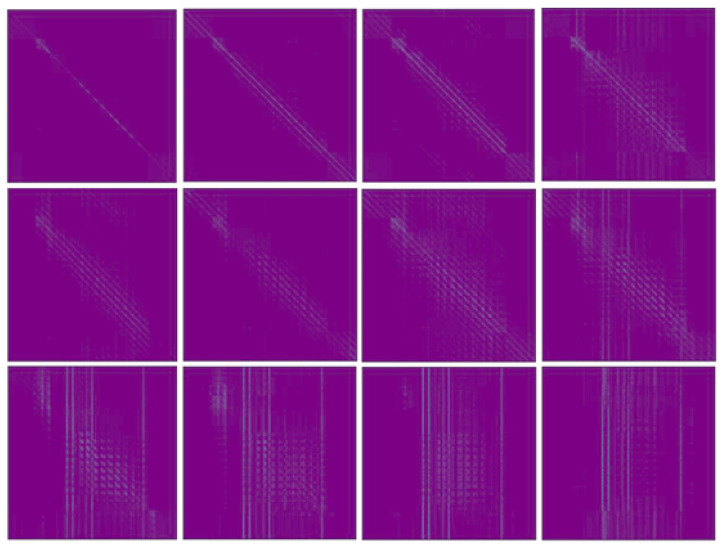
The attention maps across all 12 Transformer blocks reveal distinct evolutionary patterns in three representative cases. The first row demonstrates a consistent intensification of diagonal attention patterns with increasing layer depth, maintaining complete absence of vertical striping throughout all blocks. In contrast, the second row exhibits coordinated amplification of both diagonal and vertical patterns as layers deepen, showing the network’s parallel processing of local and global features. Most notably, the third row displays a characteristic transition where diagonal patterns systematically attenuate while vertical stripes progressively stabilize, ultimately dominating the attention distribution in higher layers.

The attention maps visualized from different transformer layers (Figure 2) reveal distinct patterns that delineate the feature learning progression. In the shallowest layers, attention maps exhibit pronounced diagonal patterns, indicating a strong local and self-referential processing bias where tokens primarily attend to themselves and their immediate neighbors. In intermediate layers (e.g., layers 2–4), these patterns evolve into diffused and branched diagonal structures, signifying an expansion of receptive fields and the integration of contextual information from a broader neighborhood. In the deepest layers, the attention converges onto a few select tokens, manifested as vertical stripes in the attention maps. This pattern indicates a reduced dependence on the query token’s own position and reflects a global, task-specific feature integration.

### 3.2. Multi-Hierarchy Feature Adapter

To fully harness the pre-trained knowledge of CLIP for open-vocabulary part segmentation, we propose a Multi-Hierarchy Feature Adapter (MHFA). The MHFA employs a progressive feature transformation mechanism that incrementally refines CLIP’s general-purpose, object-level features into discriminative, part-aware representations. As established in Section 3.1, features from different transformer layers exhibit significant differences in semantic granularity: shallow layers capture rich spatial details conducive to part localization, while deeper layers encode high-level semantic information beneficial for part recognition. By explicitly modeling and adapting these hierarchical features, the MHFA enables the coordinated optimization of multi-granularity visual representations, thereby establishing a robust feature foundation for precise part segmentation.

The input to MHFA consists of a sequence of multi-hierarchy features from HAFS, denoted as $F=[F_{1},F_{2},\ldots ,F_{L}]$, where each feature map as $F_{l}\in R^{n\times d}$ is extracted from the *l*-th layer of the CLIP’s image encoder. Here, *n* represents the number of patches (including the [CLS] token), and *d* is the feature dimension. MHFA is essentially an Adapter operation—a parameter-efficient fine-tuning technique—therefore, it maintains consistent input and output dimensions and facilitates the transfer of object features to part features. However, since the final output of the model is masks, a Repeat operation is added after the Adapter to ensure that each category has a corresponding mask. The module outputs a corresponding sequence of enriched features $A=[A_{1},A_{2},\ldots ,A_{L}]$, where each $A_{l}\in R^{E\times n\times d}$ incorporates enhanced part-level information, and *E* denotes the number of part categories. Notably, the linear layer parameters within the MHFA are shared across all feature scales, promoting generalization and efficiency. The output features $A_{l}$ are thus specifically optimized for part segmentation.

Building on the complementary nature of visual features, where shallow layers provide rich local details and deep layers encode robust global semantics, we introduce the Multi-Hierarchy Feature Adapter (MHFA) to progressively refine features from an object-level to a part-level representation. The MHFA employs linear layers to adapt general-purpose CLIP features for part-aware tasks. A key component is an α modulation factor, which selectively enhances part-specific features while preserving CLIP’s valuable prior knowledge. The MHFA process can be formally represented as follows:

(3) $$A_{l}=Repeat\left(\alpha_{1}Linear\left(Linear\left(F_{l}\right)\right)+\alpha_{2}F_{l}\right)$$

### 3.3. Text Encoder

Human communication is inherently contextual, relying on natural and situational language. For instance, a person is more likely to say, “Could you pass me the mug?” than to issue a direct action command like, “Please grab the handle of the mug.” Therefore, for effective human–robot interaction, it is essential for robots to comprehend such contextual commands and accurately identify the relevant objects and their constituent parts to execute the intended task. To bridge this semantic gap, we leverage a Large Language Model (LLM) to parse natural language instructions and extract structured information—specifically, the target objects and their associated parts. This process translates high-level human intent into actionable robotic commands. The specific prompt template designed to guide the LLM in this extraction task is detailed in Table 1.

In open-vocabulary tasks, we observe that while adapters can effectively refine image features, applying them to CLIP’s text encoder risks degrading its valuable object-level prior knowledge, as the encoder is pre-trained specifically on object-level concepts. Consequently, we preserve the integrity of CLIP’s original text encoder without introducing any fine-tuning or adapters. This design is crucial because CLIP’s contrastive learning aligns text features primarily with global, final-layer image features. As a result, these textual representations possess a strong bias towards global visual patterns and exhibit weaker correlations with local, part-level features. A direct fusion of local image features with these global text embeddings could therefore disrupt the semantic coherence of CLIP’s established alignment.

Therefore, our approach aligns the adapted multi-scale features $A_{l}$ with the text embeddings Text instead of directly aligning the original CLIP image features $F_{l}$ with the text. To facilitate this multi-hierarchy feature–text alignment, the adapted visual features $A_{l}$ are projected into a common embedding space. The text embedding extraction process can be formally described as follows, yielding a text feature matrix $T\in R^{E\times J}$, where *E* is the number of part categories and *J* is the feature dimension:

(4) T=Repeat(CLIPTextEncoder(Text))

where Text denotes the input textual prompts.

### 3.4. Hierarchical Multimodal Alignment Module

**Figure 3:**
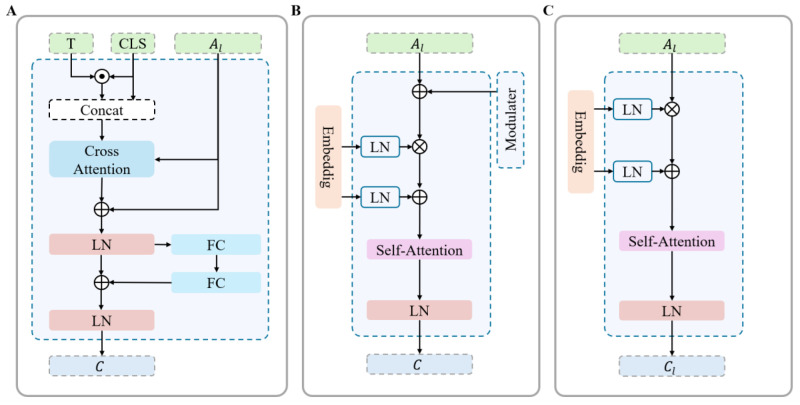
Architecture comparison of text–image feature alignment mechanisms. (**A**) ZegCLIP aligns features through cross-attention. (**B**) CLIPSeg fuses features using self-attention. (**C**) Our proposed Hierarchical Multimodal Alignment Module (HMAM).

To overcome the limitations of CLIP’s object-level priors, we propose a Hierarchical Multimodal Alignment Module (HMAM). The HMAM tackles a fundamental challenge in vision transformers: high-level features are semantically rich but spatially coarse. As shown in Figure 3, the HMAM leverages both cross-attention and self-attention mechanisms to achieve fine-grained image–text alignment. Building on our multi-level feature analysis (Section 3.1), the HMAM implements a hierarchical alignment strategy that simultaneously preserves category discriminability and improves mask coherence.

The core of HMAM is a refined alignment mechanism between textual semantics and multi-level visual features. Unlike CLIPSeg’s modular design, which directly fuses image features before cross-modal interaction, our HMAM employs a β-weighted fusion strategy to integrate textual information with both local and global image features. HMAM introduces critical improvements to this foundation. Specifically, whereas the original modular design first performs additive fusion across image feature scales and then applies cross-modal attention, our HMAM adopts a hierarchical independent alignment strategy. This approach first aligns each level of image features with text features separately through dedicated cross-attention modules, then it performs a β-weighted fusion of the resulting aligned features.

Specifically, HMAM maintains the independence of multi-level visual features, allowing each level to establish precise cross-attention mappings with text features prior to a parameterized weighted fusion. This design preserves the unique characteristics of each feature scale during cross-modal interaction, thereby achieving fine-grained alignment, minimizing feature contamination, and successfully balancing classification accuracy with mask coherence.

The cross-modal alignment in HMAM between text features T and multi-level visual features *A* is implemented as follows: (1) Element-wise multiplication and addition between projected T and A to establish preliminary correlations. (2) Deep fusion of the interacted features via a Self-Attention mechanism. (3) Normalization of the fused representations. (4) Generation of the final multimodal features $C\in R^{\left(E\times n\times k\right)}$ via a β−weighted summation of the normalized features from all levels, integrating global semantics with local details. Crucially, a linear projection (with non-shared parameters across levels) is applied to the text features *T* prior to the element-wise operations. Note that although the dimensions of *A* and *T* differ, the dimension *d* of *A* can be transformed to *J* via a Linear() operation, and the aligned features are then passed through the final LN layer in HMAM to reduce the dimension from *J* to *k*. The detailed algorithm is summarized below:

(5) $$C_{l}=LN\left(SelfAttention\left(Linear\left(A_{l}\right)⊙Linear\left(T\right)+Linear\left(T\right)\right)\right)$$

(6) $$C=\beta_{1}C_{l}+\beta_{2}C_{\left(l+1\right)}+\ldots +\beta_{L}C_{L}$$

### 3.5. Part Segmentation Generation

The final part segmentation masks must match the spatial dimensions of the input image (H×W). However, the fused multimodal features $C\in R^{\left(E\times n\times k\right)}$ have a lower resolution, necessitating a post-processing decoder to restore spatial detail. The decoder first projects and reshapes features $C^{\prime }$, which is *C* is obtained by removing the [CLS] token from C., then it applies convolutional layers to generate intermediate feature maps $M^{\prime }\in R^{\left(E\times n\times p\times q\right)}$(k=p×q+1), and finally, it employs a transposed convolution for upsampling to the target resolution. The final output $M\in R^{\left(E\times H\times W\right)}$ thus matches the input image size, where the channel dimension *E* corresponds to the number of part categories, with each channel representing the segmentation logits for a specific class. The detailed post-processing pipeline is as follows:

(7) $$M^{\prime }=Conv2D\left(Reshape\left(C^{\prime }\right)\right)$$

(8) $$M=ConvTranspose2D(ConvTranspose2D\left(M^{\prime }\right))$$

To enable effective learning for part segmentation, we optimize our model with a composite loss function that provides dual supervision at both the pixel and category levels. The overall objective L combines a mask-forming loss $L_{Mask}$ with a semantic alignment loss $L_{Class}$, formulated as:

(9) $$L=\lambda_{1}L_{Mask}+\lambda_{2}L_{Class}$$

The overall objective L has weights $\lambda_{1}=0.5$ and $\lambda_{2}=0.5$. Here, $L_{Mask}$ is the Binary Cross-Entropy with Logits Loss (BCEWithLogitsLoss), and $L_{Class}$ is the standard Cross-Entropy Loss.

## 4. Results

The effectiveness of the three proposed modules is rigorously evaluated through comprehensive quantitative and qualitative studies on zero-shot open-vocabulary part segmentation tasks.

### 4.1. Implementation Details

Our framework is built upon a pre-trained CLIP model whose image and text encoders remain frozen throughout training. Only the proposed modules—the Multi-Hierarchy Feature Adapter (MHFA) and the Hierarchical Multimodal Alignment Module (HMAM)—are trained on the part segmentation datasets (Pascal-Part and ADE20K-Part). Following the zero-shot learning paradigm, we partition the dataset into mutually exclusive sets of seen and unseen part categories for training and testing, respectively. We evaluate model performance using the mean Intersection over Union (mIoU) and the harmonic IoU (hIoU) [9]. The hIoU, which balances the performance on both seen and unseen categories, serves as our primary evaluation metric. All experiments are based on the pre-trained CLIP ViT-B/16 model pre-trained contrastively on 400 M image–text pairs, conducted on a single NVIDIA V100 GPU. The batch size was set to 16 with 512×512 as the resolution of the images. We trained the model for 30,000 iterations using a cosine annealing learning rate scheduler. The learning rate decayed from an initial value of 0.0005 to a minimum of 0.000001 over 30,000 iterations.

### 4.2. Dataset Details

Part segmentation has attracted growing research interest in recent years. Aligned with established zero-shot learning benchmarks [12], we organize a Zero-Shot dataset based on publicly available part segmentation datasets (Pascal-Part and ADE20K-Part). The model is trained on seen images from the Pascal-Part or ADE20K-Part training sets and evaluated on unseen images from both the training and test sets.

Pascal-Part Dataset: This dataset provides detailed part-level mask annotations for 20 object categories, encompassing 116 original part categories. Notably, many part labels in the original dataset contain directional indicators (e.g., “front_wheel” or “rear_wheel” for bicycles). To reduce segmentation complexity and enhance model generalizability, we consolidate these directional variants into unified categories (e.g., a single “wheel” category).

**Figure 4:**
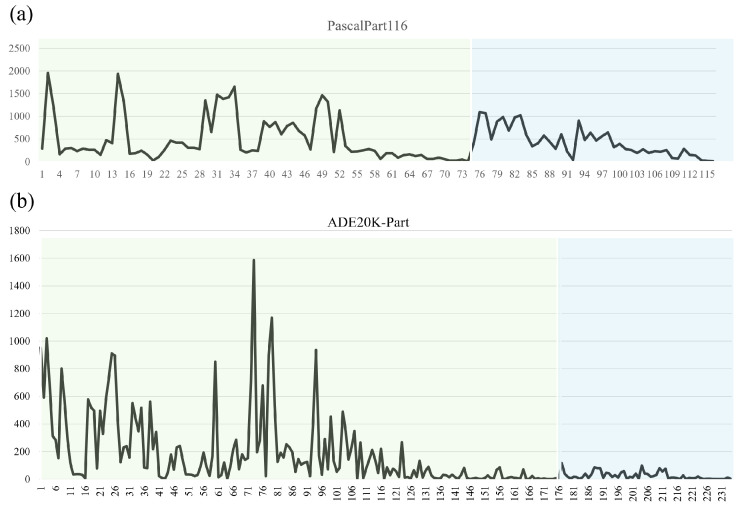
Distribution of unseen part categories in the (**a**) Pascal-Part and (**b**) ADE20K-Part test sets. The green sections represent the number of seen part categories, and the blue sections represent the number of unseen part categories.

After consolidation, the dataset comprises 8431 training and 850 test images. For our zero-shot evaluation, we partition the categories as shown in Table 2: the training set contains 15 object categories with 74 part categories (seen), while the test set contains 5 object categories with 42 part categories (unseen). Figure 4 illustrates the distribution of these unseen part categories. This consolidation strategy effectively reduces label complexity while preserving core part semantics, thereby facilitating model learning and generalization.

ADE20K-Part Dataset: This dataset offers a broader scope compared to Pascal-Part, encompassing a wider variety of object categories, with a particular emphasis on household items such as cabinets, washing machines, and chairs. We note that while the majority of objects have detailed part-level annotations, a small subset is annotated only at the instance level. These instances are systematically filtered out to ensure the integrity of our part segmentation task.

For the generalized zero-shot part segmentation task, we use 7347 images for training and 1016 for testing. The category split, detailed in Table 2, designates 33 object categories with 176 parts as seen classes for training and 11 object categories with 58 parts as unseen classes for evaluation. The distribution of these unseen part categories (Figure 4) highlights the dataset’s significant compositional diversity, presenting a robust challenge for zero-shot generalization.

### 4.3. Effect of Hierarchy-Aware Feature Selection

**Figure 5:**
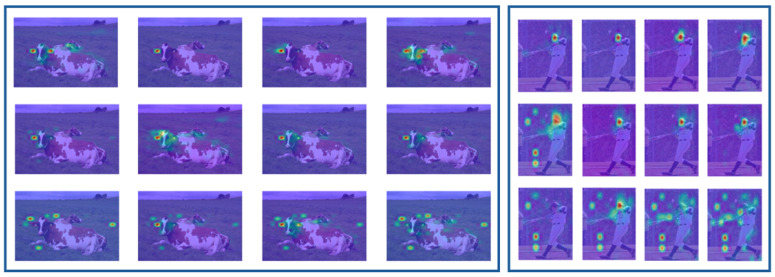
Spatial evolution of features across Transformer layers. Visualization of activation maps for representative images from ADE20K-Part and Pascal-Part datasets illustrates the progression from fine-grained details in shallow layers to broader semantics in deeper layers. The colors in the figure represent the model’s attention weights: warmer colors indicate higher attention weights, while cooler colors indicate lower attention weights.

Our analysis reveals that Transformer layers capture distinct spatial features that are crucial for part segmentation. As systematically evaluated in Table 3, the ninth layer consistently outperforms others for both seen and unseen categories. This finding challenges the conventional wisdom in object-level tasks, where final-layer features are typically dominant. Our activation analysis (Figure 5) elucidates this phenomenon: early layers preserve fine-grained spatial details that are crucial for small parts, while deeper layers develop broader contextual understanding. The ninth layer optimally balances these competing demands—local precision and global semantics. Conversely, the final layer’s excessively broad receptive field, though advantageous for object recognition, becomes detrimental in part segmentation as it often blurs boundaries between adjacent objects. These findings underscore the critical importance of task-specific layer selection over the common practice of defaulting to final-layer features. The superior performance of the ninth layer stems from its receptive field size, which aligns well with typical part scales, enabling it to preserve both precise boundaries and semantic coherence.

### 4.4. Effect of Multi-Hierarchy Feature Adapter

This study investigates multi-scale feature fusion strategies under the constraint of preserving CLIP’s frozen feature extraction pipeline. We systematically evaluate various multi-hierarchy fusion methods, with quantitative comparisons detailed in Table 4. Our proposed Multi-Hierarchy Feature Adapter (MHFA) achieves superior overall performance, particularly on the critical unseen classes and harmonic mean (hIoU) metrics across both datasets. The ablation studies confirm the contribution of each component: MHFA consistently outperforms its variants without the additive operation (denoted as MHA) or the fusion operation (MHF). It is noteworthy that while the MHF variant shows a 3.61% advantage on seen classes within ADE20K-Part, the complete MHFA framework maintains a consistent and decisive advantage in zero-shot generalization, which is the primary objective.

Further investigation into cross-modal alignment using ZegCLIP’s cross-attention mechanism (Table 4) reveals a critical dependency on the choice of visual features. We find that applying cross-attention to shallow features introduces substantial noise into the representation learning, severely degrading segmentation quality—resulting in a 17.79% performance drop on Pascal-Part and a 6.06% performance drop on ADE20K-Part compared to using the optimal ninth-layer features. This underscores that strategic layer selection is paramount; middle layers (e.g., Layer 9) uniquely provide the ideal equilibrium of spatial precision and semantic abstraction required for part segmentation.

### 4.5. Effect of Hierarchical Multimodal Alignment Module

As quantified in Table 3, features from the 9th block achieve the highest performance, closely followed by those from the 12th block. Although the 3rd and 6th block features generate superior attention maps, they contain insufficient high-level semantics. This semantic deficiency results in misclassification, ultimately compromising the segmentation accuracy. This evidences a clear trade-off: the 9th block features strike an optimal balance, while earlier blocks (3rd, 6th) offer precise localization but lack semantic richness for accurate classification, and deeper blocks (12th) possess strong categorical semantics but coarser spatial details.

Our exploration of feature combinations indicates that integrating the 9th-layer features with those from either the 3rd or 6th layer improves segmentation of seen classes, but at the cost of reduced performance on unseen classes. As shown in Table 5, the combination of features from the 3rd, 6th, and 9th layers yields the highest harmonic mean (hIoU) on both Pascal-Part and ADE20K-Part datasets. Notably, on the more complex ADE20K-Part dataset, this triple-layer combination exhibits a pronounced trade-off: it achieves the best performance for unseen classes while resulting in the lowest accuracy for seen classes. This stark contrast underscores the inherent tension between specializing to known categories and generalizing to novel ones.

We investigate feature fusion strategies within our Hierarchical Multimodal Alignment Module (HMAM) to optimally integrate multi-level features without fine-tuning CLIP. Our systematic evaluation (Table 6) identifies that a fixed-weight scheme—assigning weights of (0.5, 0.3, 0.2) to the aligned features from the 9th, 6th, and 3rd layers, respectively—yields the best performance. Ablation studies confirm the superiority of this fixed strategy over learnable weighting. As detailed in Table 6, learnable weights cause consistent performance drops: on Pascal-Part, they reduce seen-class, unseen-class, and harmonic mIoU by 2.29%, 3.71%, and 4.23%, respectively; on ADE20K-Part, unseen-class and harmonic mIoU decrease by 3.50% and 3.20%.

The effectiveness of the (0.5, 0.3, 0.2) weighting demonstrates a principled hierarchical fusion strategy: the 9th layer contributes dominant semantics, while the 6th and 3rd layers supply complementary contextual and spatial details, respectively. This finding coheres with our layer-wise feature analysis and suggests that a fixed, interpretable weighting successfully captures the intrinsic and complementary roles of different depths, thereby optimizing the fusion for part segmentation without compromising CLIP’s pre-trained representations.

### 4.6. Comparison Results

We comprehensively evaluate our method on Pascal-Part and ADE20K-Part datasets, comparing it against state-of-the-art text–image models in Table 7. Our method achieves superior performance on Pascal-Part across both seen and unseen categories, demonstrating the efficacy of its two core optimizations for part segmentation: advanced multi-scale feature fusion and hierarchical multimodal alignment.

Notably, our method outperforms the strong baseline CLIPSeg by +0.65% in unseen IoU and +0.81% hIoU for Pascal-Part, and by even larger margins of +3.26% and +2.96% on the respective metrics for the more challenging ADE20K-Part dataset. These consistent gains across datasets, although modest on Pascal-Part, still represent meaningful progress for part-aware modeling [40] and confirm the robustness and generalizability of our targeted optimizations for part segmentation.

We further compare our method with OVPart, a state-of-the-art method that fine-tunes CLIPSeg specifically for part segmentation, on both Pascal-Part and ADE20K-Part datasets (Table 7). This comparison against a strong, task-specific baseline provides a rigorous test of our method’s generalization. Following the standard zero-shot protocol, we trained and evaluated separate models for each dataset.

Our method consistently surpasses OVPart in generalizing to unseen classes, achieving performance gains of 2.61% (unseen) and 2.72% (hIoU) on Pascal-Part, and 1.59% (unseen) and 1.31% (hIoU) on ADE20K-Part. These consistent gains are significant, as they demonstrate our method’s superior generalization capability over a strong, task-specific fine-tuning approach, highlighting the effectiveness of our architectural optimizations in true zero-shot scenarios.

### 4.7. Ablation Study

Ablation studies on Pascal-Part and ADE20K-Part (Table 8) validate our core design choices for the Hierarchical Multimodal Alignment Module (HMAM) and Multi-Hierarchy Feature Adapter (MHFA), using a CLIPSeg-based setup as the baseline. The results reveal three principal findings:

First, the HMAM is crucial for accurate segmentation, underscoring the necessity of our hierarchical alignment strategy over a simple fusion of features. Second, incorporating a Text Adapter (TA) within the MHFA consistently degrades performance. We attribute this to the corruption of CLIP’s robust textual priors when fine-tuned with limited part-level data, confirming that textual representations are best left frozen. Third, and most critically, the Text Adapter (TA) alone degrades performance, whereas the Image Adapter (IA) delivers substantial performance gains, and adding HMAM on top of the Image Adapter brings further enhancements to segmentation. This validates our hypothesis that part segmentation requires a recalibration of visual features across different hierarchies, a need not adequately met by frozen object-level features. This finding directly confirms the feature selection in HAFS and underscores the fundamental difference from object-level tasks: effective part recognition depends on adaptively leveraging both the fine-grained details from shallow layers and the semantic context from deeper layers.

### 4.8. Qualitative Results

**Figure 6:**
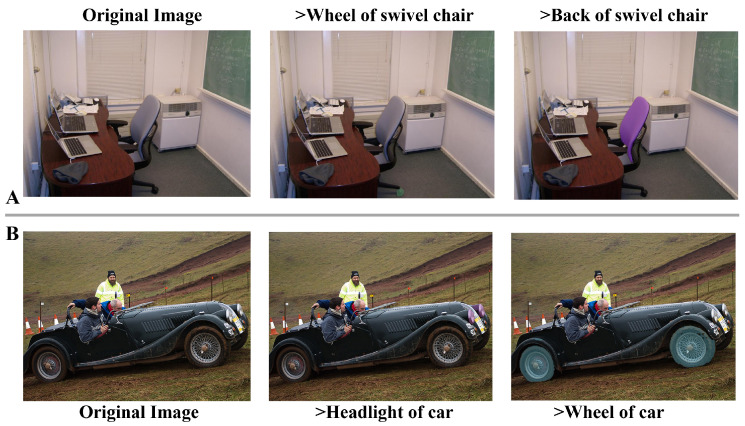
Qualitative results under different text prompts. (**A**) shows an indoor swivel-chair example, including the original image and the segmentation results under the prompts “wheel of swivel chair” and “back of swivel chair”. (**B**) shows an outdoor car example, including the original image and the segmentation results under the prompts “headlight of car” and “wheel of car”.

We perform qualitative analysis on unseen classes from the Pascal-Part and ADE20K-Part test sets to visually validate our method’s zero-shot part segmentation capability, as shown in Figure 6. The figure showcases representative examples where the model generates precise segmentation masks (colored regions) in direct response to diverse textual prompts.

These results demonstrate our model’s proficiency in interpreting natural language commands and accurately localizing the corresponding parts, even within cluttered multi-object scenes. This robust alignment between language and pixel-level perception underscores the model’s strong generalization and highlights its potential for real-world applications requiring fine-grained, language-guided segmentation.

## 5. Conclusions

The rise of embodied intelligence demands that agents advance from object-level to part-level visual understanding. However, the prowess of Visual Language Models (VLMs) in object recognition does not directly translate to part-level tasks, severely limiting robotic capabilities in complex scenarios. To bridge this gap, we propose a comprehensive framework for open-vocabulary part segmentation. Our contributions are threefold: (1) a Hierarchy-Aware Feature Selection (HAFS) mechanism that identifies optimal feature layers (3rd, 6th, 9th) balancing spatial and semantic information; (2) a Multi-Hierarchy Feature Adapter (MHFA) that progressively transforms object-level features into part-aware representations; and (3) a Hierarchical Multimodal Alignment Module (HMAM) that enables precise comprehension of part-level semantics from language instructions. Extensive experiments on standard benchmarks show that our method achieves hIoU gains of 0.81% on Pascal-Part and 2.96% on ADE20K-Part over strong baselines.

This work also reveals two key limitations: (1) a semantic gap persists as object-level text features (e.g., “door”) are misaligned with part-level visual concepts (e.g., “doorknob”); (2) current prompt engineering is inadequate for expressing intricate part hierarchies and functional relationships (e.g., between a “screwdriver handle” and its “bit”). Future work will pursue function-oriented part understanding, deeply integrating functional semantics with visual features. This direction promises to enable more human-like reasoning in agents, paving the way for embodied systems that achieve genuine part-level environmental comprehension.

## Figures and Tables

**Table 1:** The prompt structure for the LLM.

Prompt: Given an action task, output the tools used or the components of the objects touched by the action. This component refers to the part of the object that the person’s hand needs to directly touch when performing the above task.
Example: Input: Robot, could you pass me the mug? Answer: Mug, Handle Input: Robot, could you unscrew this bottle for me? Answer: Bottle, Lid
Task: Input: <instruction> Answer: <Object>, <Part>

**Table 2:** The distribution of object and part categories between seen and unseen splits in Pascal-Part and ADE20K-Part datasets.

Pascal-Part	Seen	Unseen	Total
Object	15	5	20
Part	74	42	116
ADE20K-Part	Seen	Unseen	Total
Object	33	11	44
Part	176	58	234

**Table 3:** The influence of image feature layers of different scales on component segmentation on Pascal-Part and ADE20K-Part datasets.

Dataset	Hierarchy	Seen	Unseen	hIOU
Pascal-Part	3	13.39	2.11	3.65
	6	28.03	5.08	8.59
	9	39.95	17.71	24.54
	12	35.04	9.16	14.53
ADE20K-Part	3	7.56	0.64	1.18
	6	16.79	2.19	3.87
	9	21.06	5.70	9.00
	12	17.93	4.33	6.97

**Table 4:** Comparison of different hierarchical alignment methods for part segmentation.

Dataset	Fusion	Seen	Unseen	hIOU
Pascal-Part	ZegCLIP	31.18	3.79	6.75
	MHA	38.12	6.76	11.49
	MHF	41.87	14.86	21.94
	MHFA	43.85	18.33	25.86
ADE20K-Part	ZegCLIP	18.49	1.6	2.94
	MHA	17.94	0.79	1.52
	MHF	23.84	4.62	7.74
	MHFA	20.23	9.67	13.09

**Table 5:** The impact of MHFA image features on part segmentation performance across both Pascal-Part and ADE20K-Part datasets.

Dataset	3	6	9	Seen	Unseen	hIOU
Pascal-Part	✓		✓	42.82	14.36	21.51
		✓	✓	40.18	15.9	22.78
	✓	✓	✓	43.85	18.33	25.86
ADE20K-Part	✓		✓	22.89	6.55	10.18
		✓	✓	23.31	6.69	10.40
	✓	✓	✓	20.23	9.67	13.09

✓ in the table indicate which feature layers were used.

**Table 6:** Part segmentation performance (hIoU) with different fixed weight schemes on Pascal-Part and ADE20K datasets.

Dataset	β	Seen	Unseen	hIOU
Pascal-Part	Dynamic	41.56	14.62	21.63
	(0.6,0.2,0.2)	43.46	18.29	25.75
	(0.6,0.25,0.15)	43.24	16.87	24.27
	(0.5,0.3,0.2)	43.85	18.33	25.86
ADE20K-Part	Dynamic	24.9	6.17	9.89
	(0.6,0.2,0.2)	21.81	7.34	10.98
	(0.6,0.25,0.15)	21.63	7.64	11.29
	(0.5,0.3,0.2)	20.23	9.67	13.09

**Table 7:** Comparison of the results with those of other methods based on the text–image model.

Dataset	Fusion	Seen	Unseen	hIOU
Pascal-Part	CLIPSEG	42.99	17.68	25.05
	OVPART	43.78	15.72	23.14
	Ours	43.85	18.33	25.86
ADE20K-Part	CLIPSEG	24.24	6.41	10.13
	OVPART	21.71	8.08	11.78
	Ours	20.23	9.67	13.09

**Table 8:** Ablation studies of the proposed modules on Pascal-Part and ADE20K-Part datasets.

Dataset	Model	TA	IA	HMAM	Seen	Unseen	hIOU
Pascal-Part	Baseline				42.99	17.68	25.05
	Variant			✓	41.81	17.47	24.64
		✓		✓	40.91	15.42	22.39
			✓	✓	43.85	18.33	25.86
		✓	✓	✓	42.09	15.7	22.87
ADE20K-Part	Baseline				24.24	6.41	10.13
	Variant			✓	22.79	7.02	10.73
		✓		✓	24.65	5.45	8.91
			✓	✓	20.23	9.67	13.09
		✓	✓	✓	21.55	8.30	11.99

✓ marks components that are employed.

## Data Availability

Publicly available datasets were analyzed in this study. The datasets used in this work are ADE20K-Part and Pascal-Part, as described in the cited references. No new data were generated in this study.
